# Differences in prognosis and underuse of adjuvant chemotherapy between elderly and non‐elderly patients in stage III colorectal cancer

**DOI:** 10.1002/ags3.12604

**Published:** 2022-08-26

**Authors:** Takuya Shiraishi, Hiroomi Ogawa, Ikuma Shioi, Naoya Ozawa, Katsuya Osone, Takuhisa Okada, Makoto Sohda, Ken Shirabe, Hiroshi Saeki

**Affiliations:** ^1^ Department of General Surgical Science Gunma University Graduate School of Medicine Maebashi Japan

**Keywords:** adjuvant chemotherapy, colorectal cancer, elderly patients, prognosis, sarcopenia

## Abstract

**Aim:**

We aimed to clarify the use of adjuvant chemotherapy and the prognosis of elderly colorectal cancer patients compared with non‐elderly patients, and the usefulness of sarcopenia as an indicator for the introduction and completion of adjuvant chemotherapy.

**Methods:**

Between 2013 and 2021, 215 patients with pStage III disease were included. We investigated perioperative clinicopathological factors, adjuvant chemotherapy details, and prognosis. Preoperative sarcopenia status was evaluated using computed tomography images. Elderly patients were defined as those aged ≥70 years.

**Results:**

We included 121 (56.3%) and 94 (43.7%) non‐elderly and elderly patients, respectively. Among the elderly patients, 47 had sarcopenia. There were no significant differences in the incompletion rate of adjuvant chemotherapy between elderly and non‐elderly patients (27.1%/16.2%, *P* = 0.119). The most common reason for the discontinuation of adjuvant chemotherapy was side effects, regardless of age. The respective 3‐year‐disease free survival of patients with no/completed/incomplete adjuvant chemotherapy were 65.5%, 80.2%, and 57.7% for non‐elderly patients (*P* = 0.045) and 73.4%, 70.6%, and 71.6% for elderly patients (*P* = 0.924). The number of elderly patients with sarcopenia was significantly higher in patients without adjuvant chemotherapy (*P* = 0.004) and those with incomplete adjuvant chemotherapy (*P* = 0.004). The 3‐year‐disease free survival of elderly sarcopenic patients without and with adjuvant chemotherapy were 78.3% and 59.2%, respectively (*P* = 0.833).

**Conclusion:**

Elderly patients did not show a benefit of adjuvant chemotherapy regardless of whether they had completed adjuvant chemotherapy, unlike non‐elderly patients. Moreover, the evaluation of preoperative sarcopenia in elderly colorectal cancer patients may be useful in determining the indication for adjuvant chemotherapy.

## Graphical abstract

This study analyzed data from consecutive patients with stage III colorectal cancer following curative resection and examined the reason for not introducing adjuvant chemotherapy (AC) and the differences in prognosis between completed and incomplete AC in elderly and non‐elderly patients. Furthermore, this study also looked into the usefulness of sarcopenia as an indicator for the introduction and completion of AC.

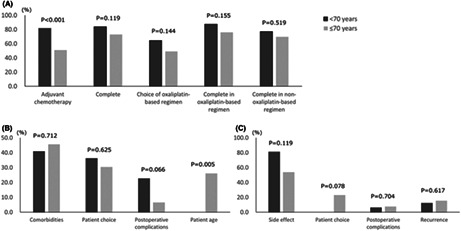

## 1 INTRODUCTION

Colorectal cancer (CRC) is one of the most common cancers in Japan and the third most common cancer worldwide. Approximately 20% of patients with CRC are diagnosed with stage III disease.^1^ A meta‐analysis showed that 5‐fluorouracil (FU)‐based postoperative adjuvant chemotherapy (AC) was associated with significant improvements in disease‐free survival (DFS) and overall survival (OS),^2^ and AC has been established as a standard treatment for stage III CRC.^3^

As the number of elderly people increases, the opportunities for treatment, including surgery and chemotherapy, for elderly patients with CRC also increase.^4^ Elderly patients have various combinations of comorbidities and geriatric syndromes and are non‐uniform.^5^ Therefore, patients older than 70 years are often excluded from several studies on the efficacy of AC, and evidence of AC in elderly patients is lacking. Additionally, the benefits of AC have not been unified in elderly patients with CRC. Subset analyses of randomized clinical trials involving oxaliplatin and capecitabine as AC showed improvements in DFS and OS in patients older than 70 years without any notable increases in toxicities.^6^, ^7^, ^8^, ^9^ Conversely, several analyses showed worse outcomes with a 5‐FU with oxaliplatin regimen in AC for patients older than 70 years than with 5‐FU alone.^10^, ^11^, ^12^ The beneficial effect of adding AC after radical surgery on recurrence and mortality in elderly patients remains controversial. Recently, retrospective studies have confirmed the survival advantages of AC in elderly CRC patients.^13^, ^14^ However, the reason for not introducing AC is unclear, and there is insufficient information on the differences in prognosis between patients with completed and incomplete AC.

To provide AC to elderly patients with CRC, clinicians need to know the prognosis with and without AC for elderly patients and the reasons for failure to introduce and complete AC. Old age and underlying diseases have been reported as reasons for not introducing AC^15^; however, there are few reports comparing the reasons for non‐completion with those of non‐elderly patients. Generally, completion of AC is effective in preventing postoperative recurrence and improving prognosis,^2^, ^3^ and in elderly patients, as in the non‐elderly, there may be an effect in reducing postoperative recurrence in patients where completion is achieved. If the reasons for failure can be clarified, AC can be safely administered to the elderly population. Guidelines recommend AC to fit elderly patients.^3^ Although the presence or absence of sarcopenia as an objective measure of fit may be helpful in determining whether AC can be initiated and completed, there are few reports on whether the presence or absence of sarcopenia affects whether AC is introduced or completed.^14^

In this study, we analyzed data from consecutive patients with stage III CRC following curative resection and examined the reasons for not introducing AC and the differences in prognosis between completed and incomplete AC in elderly and non‐elderly patients. We also clarified the usefulness of sarcopenia as an indicator for the introduction and completion of AC in elderly patients.

## 2 METHODS

### 2.1 Patients

All patients with pStage III CRC treated between July 2013 and July 2021 at Gunma University Hospital, Japan, were enrolled in this study. All patients were staged according to the American Joint Committee on Cancer 8th edition manual for CRC. The inclusion criteria were as follows: (a) proven stage III pathology and (b) radical surgery. The exclusion criteria were as follows: (a) palliative resection, (b) no proven adenocarcinoma pathology, and (c) positive pathological resection margin. This study complied with the standards of the Declaration of Helsinki and the current ethical guidelines and was approved by the Institutional Review Board of Gunma University Hospital (Approval Number: HS2021‐260).

### 2.2 Observation data

The following data were extracted from the medical and surgical reports: age, sex, body mass index (BMI), performance status (PS), comorbidities (hypertension [HT]), diabetes mellitus (DM), cardiovascular, pulmonary, and central nervous system diseases, tumor site, clinical stage, AC and its details, approach type (open or laparoscopy), surgery type (D3 lymph node dissection or not), operation time, blood loss, postoperative complications, and postoperative hospital stay.

Performance status was evaluated using Eastern Cooperative Oncology Group (ECOG)‐PS.^16^ Cardiovascular diseases included atrial fibrillation, angina pectoris, myocardial infarction, and heart failure. Pulmonary diseases included chronic obstructive pulmonary disease (COPD) and interstitial pneumonia. Central nervous system diseases included cerebral infarction, cerebral hemorrhage, dementia, and Parkinson's disease. Postoperative complications were defined as morbidities with all Clavien‐Dindo grades that occurred 30 days after surgery. Details of AC, introducing or not, the reasons for not introducing, the regimens, continuing or not, and the reasons for discontinuation were investigated. In addition, the relative dose intensity (RDI) of AC was calculated. The RDI was the proportion of the dose intensity of the standard regimen that patients received over their course of chemotherapy. For each drug within each regimen, the total dose received by the patient was divided by the total dose specified in the corresponding standard regimen, and these proportions were averaged across drugs within a given regimen.

Postoperative follow‐up included blood tests, including tumor marker measurements at 3 months, and enhanced computed tomography (CT) scans, including chest and abdomen, every 6 months. Colonoscopy was performed every 1‐2 years. Positron emission tomography was performed if recurrence was suspected, and postoperative recurrence was confirmed by clinical, histological, or serial radiological follow‐up. Patient follow‐ups were performed until February 2022.

### 2.3 Definition of elderly patients, completed or incomplete AC, and sarcopenia status

Many clinical studies have reported the clinicopathological features and prognosis of elderly patients, defined as patients over 70 years of age.^17^ Therefore, we defined 70 years of age as the threshold between the non‐elderly and elderly patients.

Patients that completed the intended course as scheduled at the time of introduction were considered part of the completed AC group. Completed AC included patients whose drug dose was reduced or treatment was postponed. Patients that did not complete the intended course as scheduled at the time of induction were considered part of the incomplete AC group.

Sarcopenia status was assessed using the psoas muscle index (PMI). As described in detail in a previous report,^18^ PMI was calculated using the psoas muscle cross‐sectional area assessed at the lower edge of the third lumbar spine (L3) divided by the square of the patient's height. The cross‐sectional area of the psoas muscle was measured by automatically outlining and manually correcting the CT images using software (Synapse Vincent; Fujifilm). Sarcopenia was defined using sex‐specific cutoff values for PMI (<524 mm^2^/m^2^ for men and <385 mm^2^/m^2^ for women), as described in a previous study.^18^

### 2.4 Statistical analysis

The clinical factors of each patient age group (<70 and ≥70 years) were analyzed. The quantitative variables are shown as medians and ranges and were compared using the Mann‐Whitney test. Categorical variables are shown as numbers and percentages and were compared using Fisher's exact test. The 3‐year DFS (3y‐DFS) and 3‐year OS (3y‐OS) were estimated using the Kaplan‐Meier method, and differences were assessed using the log‐rank test. All statistical analyses were performed using IBM SPSS Statistics (version 27.0. Armonk), with the level of statistical significance set at *P* < 0.05.

## 3 RESULTS

### 3.1 Patient characteristics

Overall, 215 patients who underwent curative resection and were diagnosed with pathological stage III colorectal adenocarcinoma were analyzed (Table 1). The median patient age was 68 years (range, 28‐94 years); 126 patients (58.6%) were men, and 89 (41.4%) were women. Of the 215 patients, 94 (43.7%) were aged ≥70 years (elderly patients), and 121 (56.3%) were aged <70 years (non‐elderly patients).

**TABLE 1:** Patients characteristics (N = 215)

Age (years), median (range)	68 (28‐94)
Sex, N (%)	
Male	126 (58.6)
Female	89 (41.4)
Body mass index (kg/m^2^), median (range)	22.2 (14.3‐40.9)
Performance status, N (%)	
0	155 (72.1)
1	45 (20.9)
2	14 (6.5)
3	1 (0.5)
Hypertension, N (%)	84 (39.1)
Diabetes mellitus, N (%)	43 (20.0)
Cardiovascular disorders, N (%)	27 (11.2)
Pulmonary diseases, N (%)	12 (5.6)
Central nervous system disease, N (%)	21 (9.8)
Tumor site, N (%)	
Right side	69 (32.1)
Left side	146 (67.9)
Preoperative treatment, N (%)	13 (6.0)
Clinical T stage, N (%)	
cT1	22 (10.2)
cT2	31 (14.4)
cT3	98 (45.6)
cT4	64 (29.8)
Clinical N stage, N (%)	
cN0	105 (48.8)
cN1	87 (40.5)
cN2	23 (10.7)
Approach type, N (%)	
Open	58 (27.0)
Laparoscopy	157 (73.0)
D3 lymph node dissection, N (%)	188 (87.4)
Operation time (minutes), median (range)	288.0 (98.0‐777.0)
Blood loss (mL), median (range)	55.0 (0.0‐3449.0)
Postoperative complications, N (%)	57 (26.5)
Postoperative stay (days), median (range)	10.0 (6.0‐210.0)

Patient and surgical characteristics, including differences between elderly and non‐elderly patients, are presented in Table 2. When compared with non‐elderly patients, a higher percentage of elderly patients had worse PS (*P* < 0.001), HT (*P* = 0.001), DM (*P* = 0.005), cardiovascular disorders (*P* = 0.010), central nervous system disease (*P* = 0.002), and right colon cancer (*P* < 0.001). A lower percentage of the elderly patients underwent laparoscopic surgery (*P* = 0.040) and AC (*P* < 0.001).

**TABLE 2:** Patient and surgical characteristics including the difference between non‐elderly (<70 years) and elderly patients (≥70 years)

	<70, N = 121	≥70, N = 94	*P* value
Sex			
Male	74 (61.2)	52 (55.3)	0.389
Female	47 (38.8)	42 (44.7)	
Body mass index (kg/m^2^)			
<22	59 (48.8)	45 (47.9)	0.897
≥22	62 (51.2)	49 (52.1)	
Performance status			
0	102 (84.3)	53 (56.4)	<0.001
1	11 (9.1)	34 (36.2)	
2	7 (5.8)	7 (7.4)	
3	1 (0.8)	0 (0.0)	
Hypertension	36 (29.8)	48 (51.1)	0.001
Diabetes mellitus	16 (13.2)	27 (28.7)	0.005
Cardiovascular disorders	9 (7.4)	18 (19.1)	0.010
Pulmonary diseases	4 (3.3)	8 (8.5)	0.099
Central nervous system disease	5 (4.1)	16 (17.0)	0.002
Tumor site			
Right side	25 (20.7)	44 (46.8)	<0.001
Left side	96 (79.3)	50 (53.2)	
Preoperative treatment			
Absence	112 (92.6)	90 (95.7)	0.331
Presence	9 (7.4)	4 (4.3)	
Clinical T stage			
cT1	14 (11.6)	8 (8.5)	0.787
cT2	19 (15.7)	12 (12.8)	
cT3	53 (43.8)	45 (47.9)	
cT4	35 (28.9)	29 (30.8)	
Clinical N stage			
Negative	62 (51.2)	43 (45.7)	0.420
Positive	59 (48.8)	51 (54.3)	
Approach type			
Open	26 (21.5)	32 (34.0)	0.040
Laparoscopy	95 (78.5)	62 (66.0)	
Surgery type			
D3 lymph node dissection	107 (88.4)	81 (86.2)	0.620
Others	14 (11.6)	13 (13.8)	
Operation time (minutes)			
<240	38 (31.4)	35 (37.2)	0.371
≥240	83 (68.6)	59 (62.8)	
Blood loss (mL)			
<100	70 (57.9)	55 (58.5)	0.923
≥100	51 (42.1)	39 (41.5)	
Postoperative complications			
Absence	86 (71.1)	72 (76.6)	0.363
Presence	35 (28.9)	22 (23.4)	
Adjuvant chemotherapy			
Absence	22 (18.2)	46 (48.9)	<0.001
Presence	99 (81.8)	48 (51.1)	

The pathological characteristics, including the differences between the elderly and non‐elderly patients, are presented in Table 3. Compared with non‐elderly patients, a higher percentage of elderly patients had poorly differentiated tumors (*P* = 0.019). No significant differences were observed in other characteristics, including pathological stage.

**TABLE 3:** Pathological characteristics including the difference between non‐elderly (<70 years) and elderly patients (≥70 years)

	<70, N = 121	≥70, N = 94	*P* value
Pathological T stage			
pT1	14 (11.6)	4 (4.3)	0.171
pT2	13 (10.7)	7 (7.4)	
pT3	68 (56.2)	63 (67.0)	
pT4	26 (21.5)	20 (21.3)	
Pathological N stage			
pN1	81 (66.9)	68 (72.3)	0.395
pN2	40 (33.1)	26 (27.7)	
Pathological stage			
pStage IIIA	26 (21.5)	10 (10.6)	0.080
pStage IIIB	70 (57.9)	66 (70.2)	
pStage IIIC	25 (20.6)	18 (19.2)	
Tumor differentiation			
Well‐ or moderate differentiation	88 (72.7)	54 (57.4)	0.019
Poor differentiation	33 (27.3)	40 (42.6)	
Ly invasion			
Absence	16 (13.2)	18 (19.1)	0.238
Presence	105 (86.8)	76 (80.9)	
V invasion			
Absence	37 (30.6)	26 (27.7)	0.641
Presence	84 (69.4)	68 (72.3)	
N invasion			
Absence	66 (54.5)	55 (58.5)	0.561
Presence	55 (45.5)	39 (41.5)	
Harvested lymph nodes			
<12	7 (5.8)	9 (9.6)	0.294
≥12	114 (94.2)	85 (90.4)	

### 3.2 Introduction, type of regimen, and continuation of AC

**FIGURE 1:**
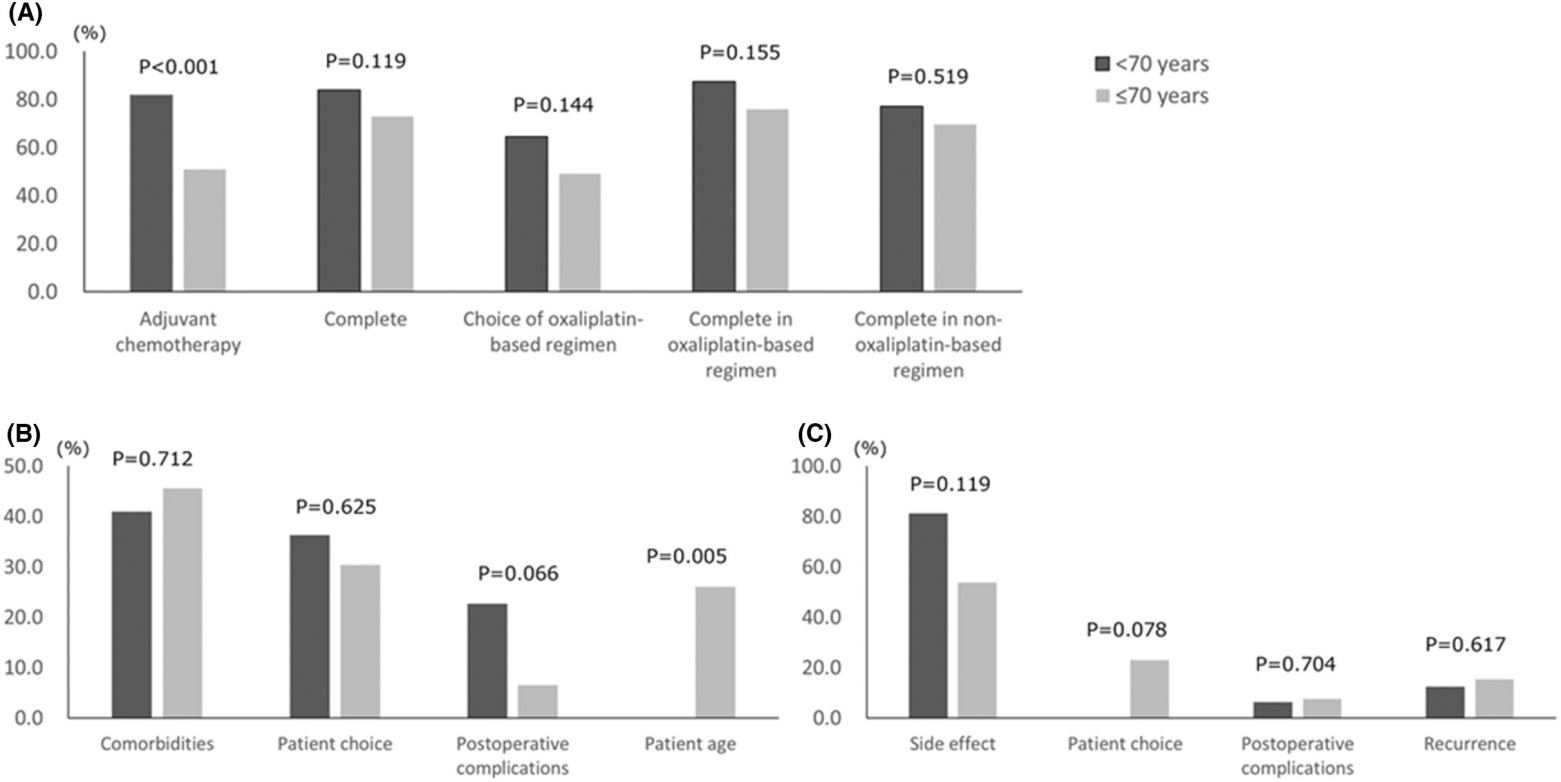
Details of adjuvant chemotherapy according to the difference between elderly (<70 years) and non‐elderly (≥70 years) patients. A, Introduction, type of regimen, and continuation of adjuvant chemotherapy. B, Reasons for not introducing adjuvant chemotherapy. C, Reasons for discontinuation of adjuvant chemotherapy.

The introduction, type of regimen, and continuation of AC in elderly and non‐elderly patients are presented in Figure 1A. Forty‐eight elderly patients (51.1%) and 99 non‐elderly patients (81.8%) received AC (*P* < 0.001). Of these patients, 13 elderly patients (27.1%) and 16 non‐elderly patients (16.2%) could not complete AC (*P* = 0.119); 25 elderly patients (49.0%) and 64 non‐elderly patients (64.5%) selected oxaliplatin‐based regimens (*P* = 0.155). Nineteen elderly (76.0%) and 56 non‐elderly patients (87.5%) completed the oxaliplatin‐based regimen (*P* = 0.155); 16 elderly (69.68%) and 27 non‐elderly patients (77.1%) completed the non‐oxaliplatin‐based regimen (*P* = 0.519).

### 3.3 Reasons for not introducing AC

Forty‐six elderly and 22 non‐elderly patients did not undergo AC after surgery. Among the elderly patients, the reasons for not introducing AC were age in 12 patients (26.1%), comorbidities in 21 (45.7%), patient choice in 14 (30.4%), and postoperative complications in three (6.5%). Among the non‐elderly patients, the reasons for not introducing AC were comorbidity in nine patients (40.9%), patient choice in eight patients (36.4%), and postoperative complications in five patients (22.7%). When compared with non‐elderly patients, a higher percentage of elderly patients had age as a reason for not introducing AC (*P* = 0.005) (Figure 1B).

### 3.4 Reasons for discontinuation of AC

Thirteen elderly and 16 non‐elderly patients were unable to complete AC. Of the patients who received AC, discontinuation of AC within three courses was observed in nine patients in the elderly (69.2%) and 12 patients in the non‐elderly group (75.0%). Among the elderly patients, the reasons for discontinuation of AC were side effects in seven patients (53.8%), recurrence in two patients (15.4%), patient choice in three patients (23.1%), and late postoperative complications in one patient (7.7%), and no emergency hospitalization during treatment. Among the non‐elderly patients, the reasons for discontinuation of AC were side effects in 13 patients (81.3%), recurrence in two patients (12.5%), and late postoperative complications in one patient (6.3%); six patients needed emergency hospitalization during AC. Side effects were the most common reason for not completing AC for both the elderly and non‐elderly groups, and no significant differences were found in the reasons for not completing AC (Figure 1C).

### 3.5 RDI by completed and incomplete AC

In all patients who were introduced to AC, the RDI for the completed AC was 100.0% and that for the incomplete AC was 30.3% (*P* < 0.001). For elderly patients, the RDI for the completed AC was 97.1% and that for the incomplete AC was 50.0% (*P* < 0.001). For non‐elderly patients, the RDI for the completed AC was 100.0% and that for the incomplete AC was 22.5% (*P* < 0.001).

### 3.6 Prognosis by induction and completion of AC

**FIGURE 2:**
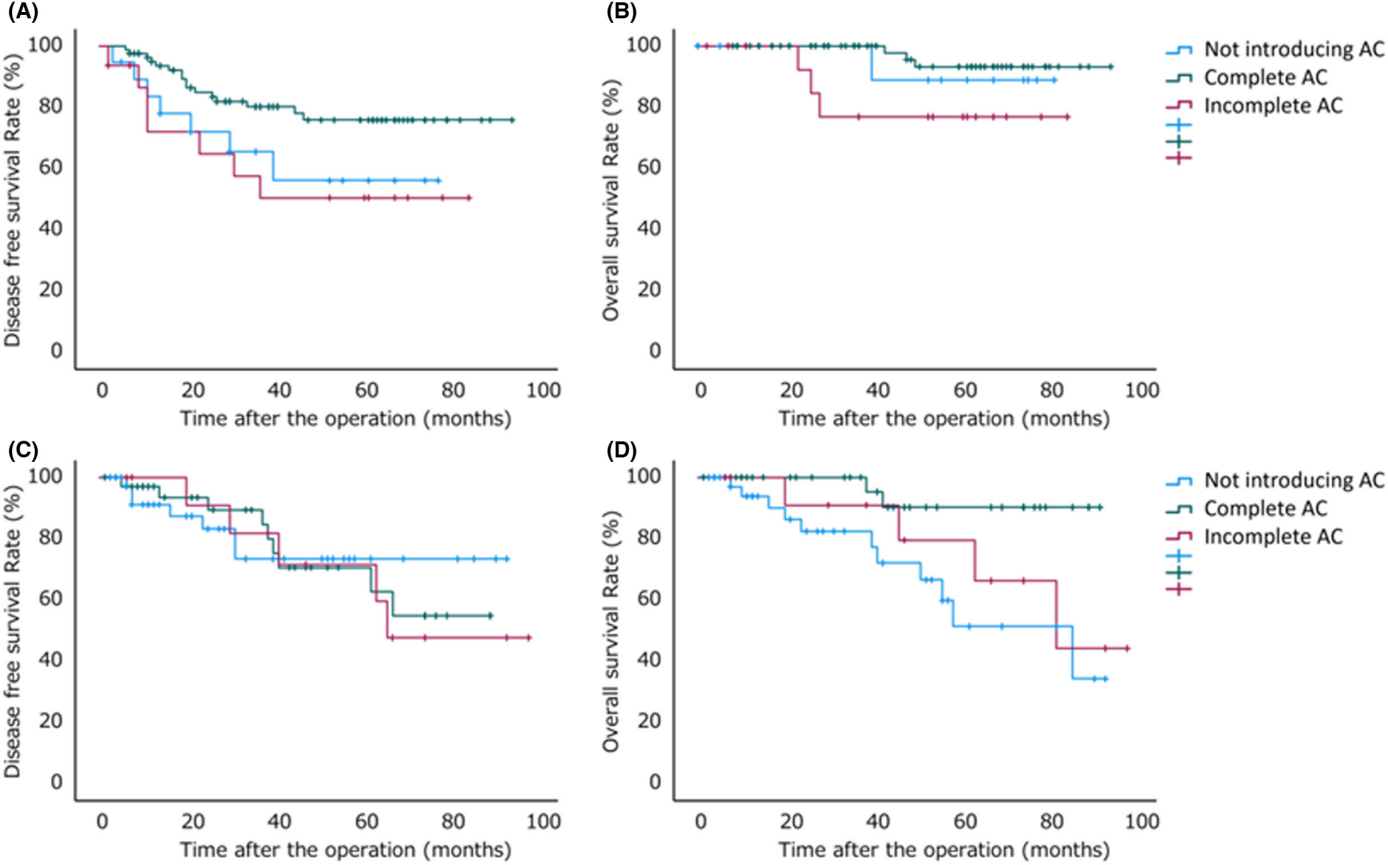
Prognosis by induction and completion of adjuvant chemotherapy. A, the 3‐year disease‐free survival (3y‐DFS) for non‐elderly patients was 65.5% for patients without adjuvant chemotherapy (AC), 57.7% for patients who had not completed AC, and 80.2% for patients who had completed AC, with a significant difference (*P* = 0.045). B, the 3‐year overall survival (3y‐OS) for non‐elderly patients was 100.0% for patients without AC, 76.9% for patients who had not completed AC, and 100.0% for patients who had completed AC, with no significant difference (*P* = 0.086). C, the 3y‐DFS for elderly patients was 73.4% for patients without AC, 71.6% for patients who had not completed AC, and 70.6% for patients who had completed AC, with no significant difference (*P* = 0.924). D, the 3y‐OS for elderly patients was 72.1% for patients without AC, 90.9% for patients who had not completed AC, and 90.2% for patients who had completed AC, with a significant difference (*P* = 0.017).

The 3y‐DFS and ‐OS were 73.6% and 91.3%, respectively, and the median follow‐up period was 37 months (range, 0–87 months). The 3y‐DFS for non‐elderly patients was 65.5% for patients without AC, 57.7% for patients who did not complete AC, and 80.2% for patients who had completed AC, with a significant difference (*P* = 0.045) (Figure 2A). The 3y‐OS for non‐elderly patients was 100.0% for patients without AC, 76.9% for patients who had not completed AC, and 100.0% for patients who had completed AC, with no significant difference (*P* = 0.086) (Figure 2B). The 3y‐DFS for elderly patients was 73.4% for patients without AC, 71.6% for patients who had not completed AC, and 70.6% for patients who had completed AC, with no significant difference (*P* = 0.924) (Figure 2C). The 3y‐OS for elderly patients was 72.1% for patients without AC, 90.9% for patients who had not completed AC, and 90.2% for patients who had completed AC, with a significant difference (*P* = 0.017) (Figure 2D). Although non‐elderly patients who completed AC showed a significant reduction in recurrence compared to the other patients, there was no significant effect of AC or completion of AC on the prevention of recurrence in elderly patients. However, OS was better in elderly patients who were introduced to AC.

### 3.7 Uni‐ and multivariate analyses for OS in elderly patients

Table 4 shows the univariate and multivariate analyses of risk factors for OS using the Cox regression model. Univariate analysis revealed that OS was associated with postoperative complications (*P* = 0.039), pathological N stage (*P* = 0.044), and AC (*P* = 0.019). The independent prognostic factors identified in the multivariate analysis were pathological N stage (HR, 6.794; 95% CI, 1.957–23.588; *P* = 0.003) and AC (HR, 0.156; 95% CI, 0.0042–0.584; *P* = 0.006).

**TABLE 4:** Uni‐ and multivariate analyses for OS in elderly patients (≥70 years)

	Univariate analysis		Multivariate analysis	
	HR (95% CI)	*P* value	HR (95% CI)	*P* value
Sex				
Male	1	0.951		
Female	1.032 (0.374‐2.852)			
Tumor site				
Right side	1	0.420		
Left side	1.489 (0.565‐3.922)			
Sarcopenia				
Absence	1	0.946		
Presence	1.033 (0.397‐2.692)			
Surgery type				
D3 lymph node dissection	1	0.545		
Others	0.631 (0.142‐2.800)			
Postoperative complications				
Absence	1	0.039	1	0.368
Presence	2.805 (1.053‐7.468)		1.620 (0.567‐4.632)	
Pathological T stage				
pT1‐3	1	0.829		
pT4	1.148 (0.328‐4.018)			
Pathological N stage				
pN1	1	0.044	1	0.003
pN2	2.707 (1.026‐7.143)		6.794 (1.957‐23.588)	
Tumor differentiation				
Well‐ or moderate differentiation	1	0.598		
Poor differentiation	1.297 (0.493‐3.416)			
Ly invasion				
Absence	1	0.344		
Presence	0.538 (0.149‐1.944)			
V invasion				
Absence	1	0.783		
Presence	1.171 (0.381‐3.602)			
N invasion				
Absence	1	0.095		
Presence	2.272 (0.866‐5.959)			
Harvested lymph nodes				
<12	1	0.580		
≥12	0.565 (0.075‐4.270)			
Adjuvant chemotherapy				
Absence	1	0.019	1	0.006
Presence	0.300 (0.110‐0.817)		0.156 (0.042‐0.584)	

### 3.8 Relationship between sarcopenia and AC in elderly patients

**FIGURE 3:**
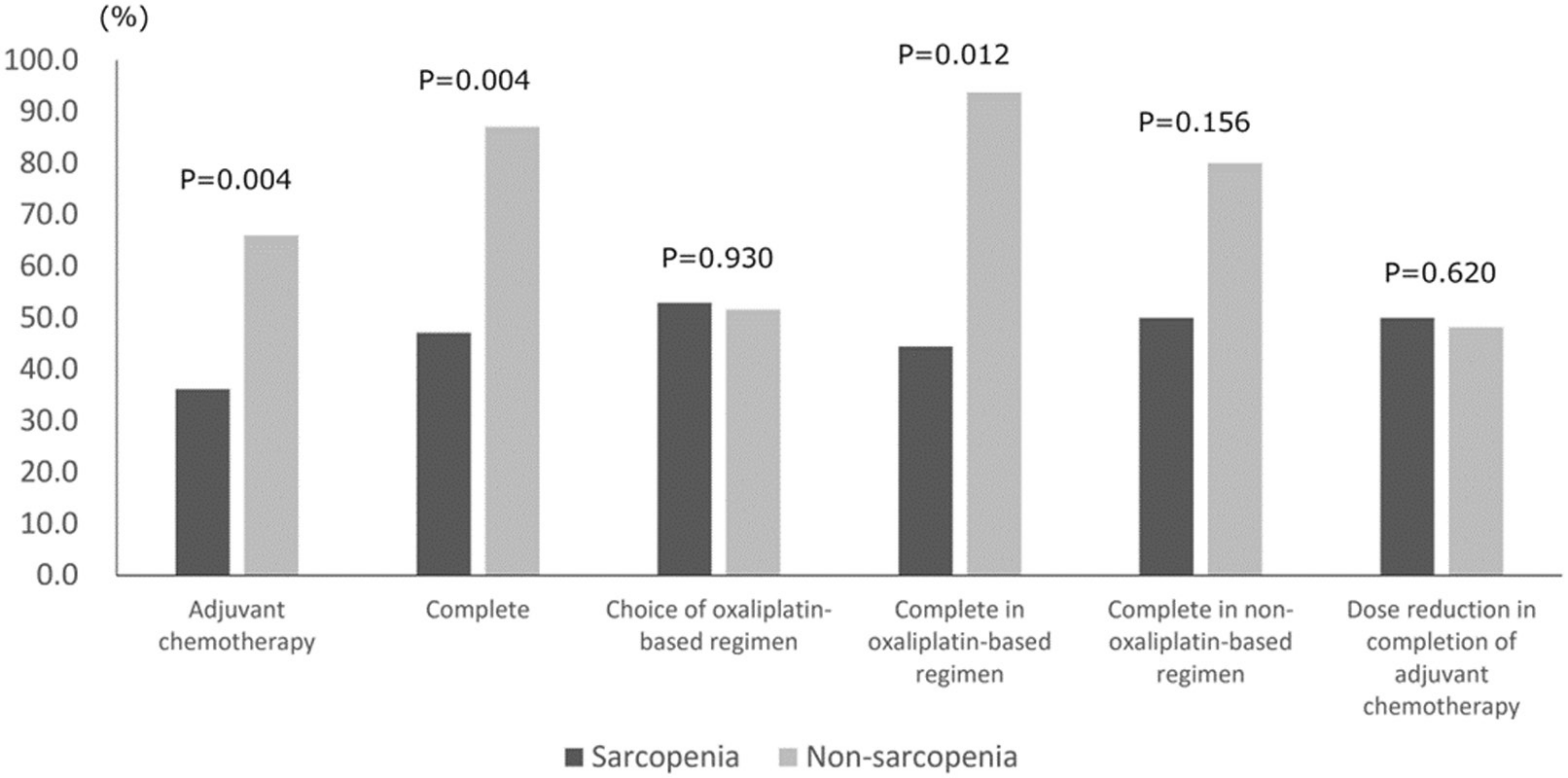
Relationship between sarcopenia and adjuvant chemotherapy in elderly patients

Among the elderly patients, 47 (50.0%) had sarcopenia. Seventeen patients with sarcopenia (36.2%) and 31 patients without sarcopenia (66.0%) underwent AC after surgery (*P* = 0.004). Among the patients who were introduced to AC, eight patients with sarcopenia (47.1%) and 27 patients without sarcopenia (87.1%) had completed AC (*P* = 0.004). There was no significant difference between patients with and without sarcopenia in the choice of oxaliplatin‐based regimens (*P* = 0.930); however, the number of patients with sarcopenia who completed oxaliplatin‐based regimens was significantly lower than that of patients without sarcopenia (*P* = 0.012). In contrast, there was no significant difference between patients with and without sarcopenia in the completion of the non‐oxaliplatin‐based regimen (*P* = 0.156) and dose reduction in the completion of AC (*P* = 0.620) (Figure 3).

### 3.9 Prognosis by induction and completion of AC

**FIGURE 4:**
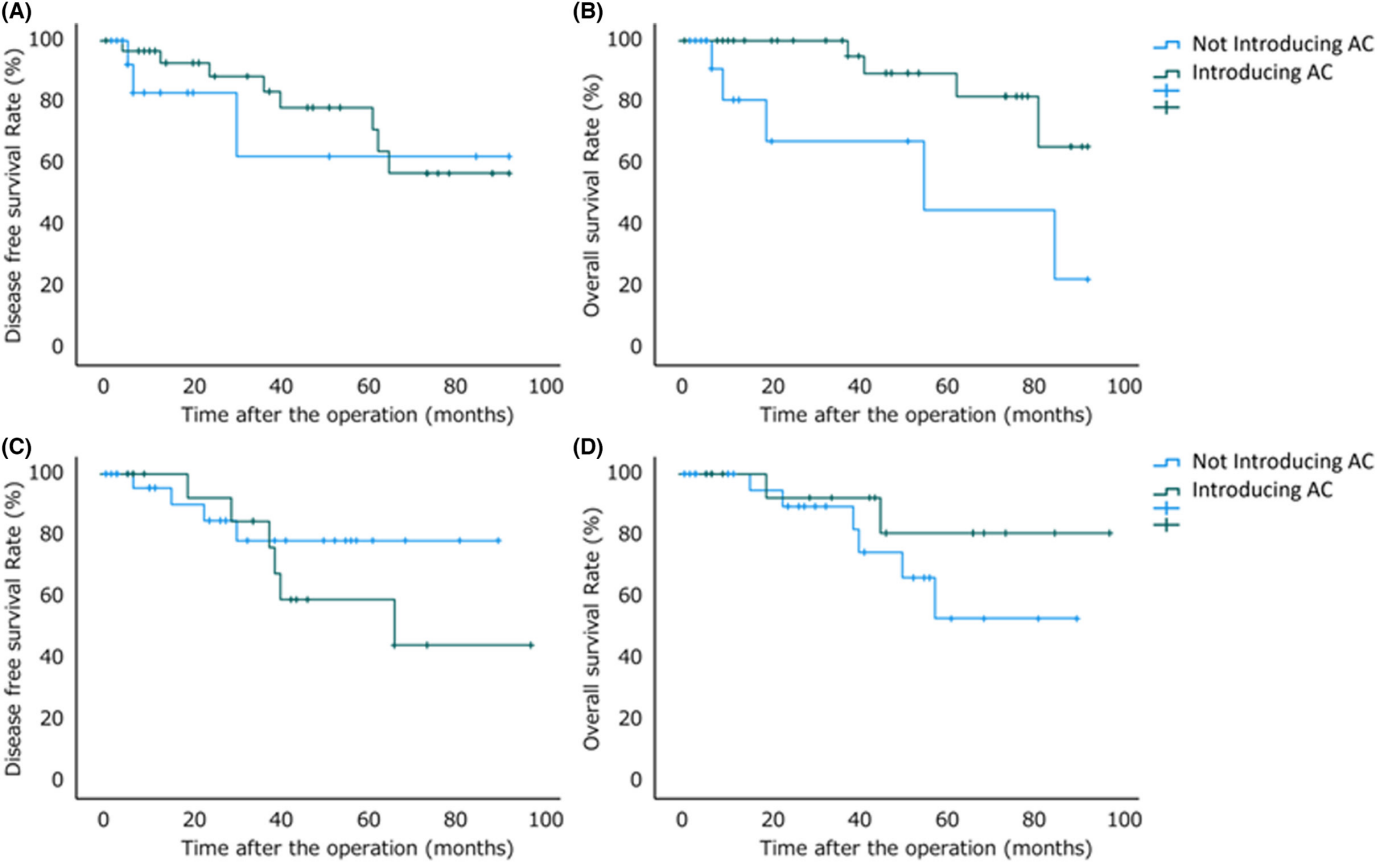
Prognosis by induction and completion of adjuvant chemotherapy. A, the 3‐year disease‐free survival (3y‐DFS) for elderly patients without sarcopenia was 62.3% for patients without adjuvant chemotherapy (AC) and 78.3% for patients with AC, with no significant difference (*P* = 0.565). B, the 3‐year overall survival (3y‐OS) for elderly patients without sarcopenia was 67.3% for patients without AC and 89.4% for patients with AC, with a significant difference (*P* = 0.016). C, the 3y‐DFS for elderly patients with sarcopenia was 78.3% for patients without AC and 59.2% for patients with AC, with no significant difference (*P* = 0.833). D, the 3y‐OS for elderly patients with sarcopenia was 74.6% for patients without AC, 92.3% for patients with AC, with no significant difference (*P* = 0.261).

The 3y‐DFS for elderly patients without sarcopenia was 62.3% for those without AC and 78.3% for those with AC, with no significant difference (*P* = 0.565) (Figure 4A). The 3y‐OS for elderly patients without sarcopenia was 67.3% for patients without AC and 89.4% for patients with AC, with a significant difference (*P* = 0.016) (Figure 4B). The 3y‐DFS for elderly patients with sarcopenia was 78.3% for patients without AC and 59.2% for patients with AC, with no significant difference (*P* = 0.833) (Figure 4C). The 3y‐OS of elderly patients with sarcopenia was 74.6% for patients without AC and 92.3% for patients with AC, with no significant difference (*P* = 0.261) (Figure 4D). AC tended to have a better prognosis in elderly patients without sarcopenia.

## 4 DISCUSSION

The introduction of AC was significantly lower in the elderly patients than in the non‐elderly patients. A meta‐analysis reported that the incidence of postoperative complications, which cause avoidance of postoperative AC, was slightly higher in elderly patients than in non‐elderly patients.^17^ Here, elderly patients had more comorbidities than non‐elderly patients; however, the proportion of postoperative complications was similar in elderly and non‐elderly patients. Moreover, the main factor for not introducing AC in elderly patients was older age. Although several studies have reported that the incidence of AC toxicity did not increase among elderly patients with CRC, patient age influenced the introduction of AC.^19^ In previous reports, the proportion of patients who did not receive AC because of their older age was high^20^; older age was also a reason for discontinuing AC.^13^ Older age is an important factor for the introduction and continuation of AC for many clinicians and patients.

There were no significant differences in the completion rate of AC between elderly and non‐elderly patients with or without oxaliplatin, and the most common reason for the discontinuation of AC was side effects, regardless of age. In addition, our results were not associated with higher hospitalization rates or toxicity in elderly patients. Elderly patients included in this study may not have continued AC until they experienced serious side effects that required hospitalization. Previous studies have reported an association between elderly patients and a higher hospitalization risk and highlighted the heightened toxicity risks associated with chemotherapy in elderly patients.^21^, ^22^ Moreover, hospitalization during AC leads to poor overall survival. Behind the need for hospitalization, patients have characteristics such as older age and higher comorbidity, and if patients have a history of hospitalization during chemotherapy, clinicians cannot potentially select effective salvage chemotherapy at the time of recurrences.^15^ To achieve complete AC, it is necessary to take proper measures against side effects regardless of age and to identify indicators such as the ability to tolerate AC.

In non‐elderly patients, the recurrence rate was low when AC was completed. In contrast, for elderly patients, the recurrence rate was similar regardless of whether AC was introduced or completed. Although several reports have shown that AC improves prognosis in elderly patients as well as in non‐elderly patients,^6^, ^7^, ^8^, ^9^ the percentage of AC completion was unknown. In general, a higher percentage of elderly patients are unable to introduce or complete AC due to multiple comorbidities.^15^ Similarly, in this study, the induction rate of AC in elderly patients was low, and the completion rate tended to be low. The effect of AC in reducing postoperative recurrences could be expected for non‐elderly patients; however, over 70 years of age did not show the benefits of AC for pStage III patients after radical resection, even after completing AC.

The proportions of elderly and non‐elderly patients who selected oxaliplatin‐based regimens were similar in the present study. The beneficial effect on recurrence and mortality of adding oxaliplatin to fluoropyrimidine‐based chemotherapies in elderly patients is controversial.^10^ The ACCENT study outcome did not suggest the benefit of adding oxaliplatin to 5‐FU in terms of DFS and OS in patients aged ≥70 years.^10^ A systematic review also reported that adding oxaliplatin in patients older than 70 years showed no benefit and only greater levels of toxicity.^23^ Currently, AC in elderly patients is not evidence‐based^24^ because there are no randomized trials performed specifically in elderly patients to reveal the benefits of prognosis and the increased risks of toxicity of AC in elderly patients. Moreover, a recent review also reported that although the benefits of AC after CRC surgery in elderly patients are similar to those in non‐elderly patients, adding oxaliplatin to a fluoropyrimidine may not be beneficial in patients above a biological age of approximately 70 years.^25^ Herein, the concomitant use of oxaliplatin may have contributed to the lack of benefits of AC.

Among elderly patients, sarcopenia discouraged the introduction of AC and decreased the completion rate of AC. Moreover, no benefit of AC was observed in patients with sarcopenia. This is because patients with sarcopenia have biologically weaker physiological functions, and their short‐term and long‐term outcomes are poorer than those of patients without sarcopenia.^26^ Patients with sarcopenia have been reported to be more prone to side effects,^27^ and clinicians need to take care of the introduction and continuation of AC for elderly patients, including many patients with sarcopenia. The efficacy of AC in elderly patients is unclear; therefore, safe AC should be provided, and sarcopenia may be useful as an indicator of the introduction and continuation of AC in elderly patients.

In patients over 70 years of age, OS tended to be better with AC in non‐sarcopenia patients; however, DFS was similar with or without AC, and our results did not provide a clear conclusion on the benefit of AC in non‐sarcopenic patients. In the multivariate analysis in patients over 70 years of age, not introducing AC was a risk factor associated with OS, but sarcopenia itself was not associated with OS. Based on the results of our study, including the survival curves, AC may improve OS in patients without sarcopenia; however, it may not improve OS in patients with sarcopenia. These results suggest that non‐sarcopenic elderly patients selected for AC generally have a better prognosis. The presence of sarcopenia before AC has been associated with a poor prognosis compared with the absence of sarcopenia.^28^ This study also showed no benefit of AC in patients with sarcopenia. In contrast, non‐sarcopenic elderly patients have found AC as useful as non‐elderly patients.^28^ AC is not recommended for sarcopenia in the elderly and may be acceptable for patients without sarcopenia; however, its benefits may be limited. In addition, oxaliplatin is not be a better choice for elderly patients with sarcopenia because the complete proportion of oxaliplatin‐based regimens was significantly low in patients with sarcopenia.

Our study has several limitations. First, this study was retrospective in nature and included a small sample size of patients with CRC from a single institution. There may have been a selection bias by including patients that were not introduced to AC due to old age. Therefore, our cohort was neither random nor unselected, and it differs somewhat from the national population of elderly CRC patients; our findings may lack generalizability. Second, our study did not include data on the expression of mismatch repair proteins (MMR). MMR expression could not be examined because MMR was uncommon in our cohort. Deficiencies in the expression of MMR tumors are more common in elderly patients,^29^ and the need and effect of AC in elderly patients may be less because the tumors are associated with a low risk of recurrence and a poor response to fluoropyrimidines.^30^ Third, about half of the patients were sensors in 3y‐DFS and 3y‐OS. These survival rates should be interpreted carefully, as the results may include patients that have not been adequately followed up. Despite these limitations, we believe that our study provides useful information regarding AC in elderly patients. First, our study suggests that AC is not recommended for elderly patients with sarcopenia and may be acceptable for patients without sarcopenia; however, the benefits may be limited. Second, our study suggests that oxaliplatin may not be better suited to elderly patients with sarcopenia.

In conclusion, we found that the introduction of AC was significantly lower in elderly patients due to older age, there were no significant differences in the completion rate of AC between elderly and non‐elderly patients, and the most common reason for the discontinuation of AC was side effects, regardless of age. In addition, the non‐elderly patients showed a benefit of AC in preventing recurrence, while the elderly patients did not show a benefit of AC regardless of whether they had induction or completion of AC. Moreover, evaluation of preoperative sarcopenia in elderly CRC patients may be useful not only for predicting postoperative prognosis, but also for determining the indication for AC.

## DISCLOSURE

Funding: The authors have not received financial support from any funding sources for this study.

Conflict of Interest: Author KS is an editorial board member of *Annals of Gastroenterological Surgery*. The other authors declare no conflict of interest for this article.

Author Contributions: TS collected and evaluated data, wrote the manuscript, and prepared figures. HO, IS, NO, CK, YS, KO, TO, MS, KS, and HS revised the manuscript and provided comments on the structure and details of the article. All authors read and approved the final manuscript.

Ethics statement: The study protocol was approved by the Institutional Review Board of Gunma University Hospital (approval no. HS2021‐260). The requirement for informed consent was waived because the analysis was based on retrospective record review.
